# Socioeconomic and demographic determinants of birth weight in southern rural Ghana: evidence from Dodowa Health and Demographic Surveillance System

**DOI:** 10.1186/s12884-016-0956-2

**Published:** 2016-07-15

**Authors:** Alfred Kwesi Manyeh, Vida Kukula, Gabriel Odonkor, Rosemond Akepene Ekey, Alexander Adjei, Solomon Narh-Bana, David Etsey Akpakli, Margaret Gyapong

**Affiliations:** Dodowa Health Research Centre, P. O. Box. DD1, Dodowa, Ghana; Shai-Osudoku District Hospital, Dodowa, Ghana; Ghana Health Services, Accra, Ghana

**Keywords:** Birth weight, Socioeconomic, Determinants, Health and demographic surveillance system, Dodowa, Ghana

## Abstract

**Background:**

Low birth weight (LBW) is one of the major factors affecting child morbidity and mortality worldwide. It also results in substantial costs to the health sector and imposes a significant burden on the society as a whole. This study seeks to investigate the determinants of low birth weight and the incidence of LBW in southern rural Ghana.

**Methods:**

Pregnancy, birth, demographic and socioeconomic information of 6777 mothers who gave birth in 2011, 2012, and 2013 and information on their babies were extracted from a database. The database of Dodowa Health and Demographic Surveillance System is a longitudinal follow-up of over 24,000 households. The incidence of LBW was calculated and the univariable and multivariable associations between exposure variables and outcome were explored using logistic regression. STATA 11 was used for the analyses.

**Result:**

The results revealed that 40.21 % of the infants were not weighed at birth and the incidence of LBW for 2011 to 2013 was 8.72, 7.04 and 7.52 % respectively. Women aged 20–24, 25–29, 30–34 years were more than twice more likely to have babies weighing ≥2.5 kg compared to those <20 years (OR:2.32, 95 % CI:1.65–3.26, OR:2.73, 95 % CI:1.96–3.79, OR:2.87, 95 % CI:2.06–4.01) and mothers who were >34 years were more than three times more likely to have babies weighed ≥2.5 kg (OR: 3.59, 95 % CI:2.56–5.04). Mothers who were civil servants were 77 % more likely to have babies weighed ≥2.5 kg (OR: 1.77, 95 % CI: 1.99–2.87) compared to those who were unemployed. After adjusting for other explanation variables, mothers from poorer households were 30 % more likely to have babies who weighed ≥2.5 kg (OR: 1.30, 95 % CI: 1.01–1.66) compared to those from the poorest households. Women with parity2 and parity > 3 were 30 % and 81 % more likely to have babies weighing ≥2.5 kg (OR: 1.30, 95 % CI: 1.03–1.63, OR: 1.81, 95 % CI: 1.38–2.35) compared to those with parity1. Male infants were 52 % more likely to weigh ≥2.5 kg at birth (OR: 1.52, 95 % CI: 1.32–1.76) compared to females.

**Conclusion:**

Our study revealed that having infant birth weight ≥ 2.5 kg is highly associated with socioeconomic status of women household, the gender of an infant, parity, occupation and maternal age.

## Background

Low birth weight (LBW) is a major public health problem worldwide especially in low and middle income countries. It is a major determinant of mortality, morbidity and disability in neonatal, infancy and childhood and has a long term impact on health outcomes in adult life. Low birth weight results in substantial costs to the health sector and imposes a significant burden the society as a whole [1].

LBW is considered the single most important predictor of infant death within the first month of delivery [2, 3] and together with preterm births, they are indicators of potential lifelong consequences to individuals, families, and communities at large. Birth weight is a good indicator of the reproductive and general health status of a population. It is not only about the baby’s health and nutritional status but also the physical and psychosocial growth and development of babies and their chances of survival [2–4].

The Centre for Disease and Control (CDC) classified birth weight as extremely low birth weight (ELBW) in infants whose birth weight was below 1 kg, very low birth weight (VLBW) in infants whose birth weight was below 1.5 kg, low birth weight (LBW) in infants whose birth weight was below 2.5 kg, normal birth weight (NBW) in infants whose birth weight was below 4 kg, and high birth weight (HBW) in infants whose birth weight was more than 4 kg Health risk depends on these classifications [5]. It was a goal of the 2012 World Health Assembly to reduce the number of infants born weighing below 2.5 kg by 30 % by the year 2025. This would translate into a 3 % relative reduction per year between 2012 and 2025 and a reduction from approximately 20 million to 14 million infants with low weight at birth [6]. The prevalence of LBW is estimated to be 15 % worldwide with a range of 3.3–38 % and occurs mostly in low and middle income countries [1] representing more than 20 million of all births per year. The prevalence is highest in South-Central Asia and sub-Saharan Africa, but there are intra-country variations. It is a global concern, as some developed countries such as Spain, Great Britain, Northern Ireland and the United States of America are also faced with high rates for their contexts [7].

Factors associated with birth weight operate broadly through genetic, socio-demographic and environmental channels. These factors include the sex of the child for the same gestational age [8]; maternal age [9, 10]; maternal birth weight [11] and maternal weight [1, 12–15]. Others include maternal nutrition - cumulatively, and during pregnancy [16, 17]; smoking [1, 18]; type of cooking fuel [19–22]; and socioeconomic status [23, 24]. While LBW in the developing world stems primarily from the mother’s poor health and nutrition, cigarette smoking during pregnancy is the leading cause of LBW in the developed world. In both developed and developing countries alike, LBW is most frequently associated with teenagers who give birth when their own bodies have not yet fully developed [25].. Studies show that in sub-Saharan Africa and other developing parts of the world, poverty, low education, late initiation of obstetric care, poor nutrition, and micronutrient supplementation during pregnancy are associated with LBW [17, 26–29]. Parity and birth intervals are also risk factors [30–35].

The Ghana Multiple Indicator Cluster Survey (MICS) found a LBW prevalence of 9.1 % and 11 % in 2006 and 2011 respectively [4, 36]. However, in 2006 only two in five babies were weighed at birth. The report shows that 54 % of infants were weighed at birth [4] with regional variations from as high as 85 % in Greater Accra region and as low as 25 % in the Northern region. Also, children from rural households and those from the poorest households are less likely than the more advantaged children to be weighed at birth [4]. Additionally, the possibility that children are weighed at birth increased with an increase in the mother’s level of education [4]. Another study in 2009 revealed that approximately 10 % of all births in Ghana were LBW [36]. Studying the socioeconomic and demographic determinants of birth weight is important for both public health and clinical perspectives since such information would be crucial in understanding the effect of demographic variables and changes in socioeconomic status of people on the birth weight of infants. In Ghana, no study has been done using longitudinal population-based data to assess the socioeconomic and demographic determinants of birth weight. This study sought to investigate the determinants of birth weight and the incidence of LBW in southern rural Ghana using population-based longitudinal data.

## Methods

### Study area

Data for this study were extracted from the Dodowa Health and Demographic Surveillance System (DHDSS) site database. The DHDSS is located in the south-eastern part of Ghana and operates within the boundaries of the Shai-Osudoku and Ningo-Prampram districts [37]. The DHDSS site lies between latitude 5° 45′ south and 6° 05′ north and longitude 0° 05′ east and 0° 20′ west with a land area of 1528.9 km^2^. It is about 41 km from the national capital, Accra [37, 38]. The two districts are made up of a population of 115,754 people in 380 communities. There are 23,647 households living in a total land area of 1442 km^2^. The inhabitants are predominantly subsistence farmers, fishermen and petty traders [38]. Road networks in the DHDSS are usually inaccessible during the wet seasons, making access to health and other services a challenge.

The DHDSS visits every household in the demographic surveillance area twice in a year to collect data on demographic, migration and other health indicators [38]. Health care services in the DHDSS are delivered by hospitals, health centres, CHPS zones, private facilities, clinics, maternity homes, mission clinics and quasi government clinics.

### Study population and sample

The study population is made up of all babies born to resident women in the DHDSS and the study sample comprised 6777 babies born to women who were resident in the DHDSS from 1st January 2011 to 31st December 2013. All babies born to women who were not resident members of the DHDSS and those born outside the study period were excluded.

### Outcome and exposure variables

The outcome variable for this study is birth weight which is binary recorded as: 1 “Birth Weigh <2.5 kg” and 0“Birth Weight ≥2.5 kg”. From the available data, eleven exposure variables were selected based on biological plausibility, the available literature and the potential to influence birth weight. These exposure variables include: infant’s gender, maternal age, maternal education, maternal occupation, parity and the intake of Intermittent Preventive Treatment (IPTp) for prevention of malaria in pregnancy. Others include whether this is the mothers first live birth or not, her marital status, antenatal (ANC) attendance, type of cooking fuel, and the wealth index.

The wealth index (socioeconomic status) is a proxy measure of a household’s long term standard of living; it is based on social status, asset ownership, and availability of utilities, among others. The index measures were combined into a wealth index, using weights derived through principal component analysis (PCA) [39]. The proxies from the PCA were divided into five quintiles; poorest, very poor, poor, less poor and least poor. Maternal ages at delivery were calculated using the mothers’ and babies’ birthdates.

### Measurement and statistical analysis

The study used secondary data from the DHDSS. Birth weight variable which was captured from the health record book of the child during the DHDSS data collection period and exposure variables were extracted from the database of DHDSS. The associations between each exposure variable and birth weight were explored at the univariable level and those significant at *p* < 0.05 were entered together into a multiple logistic regression model.

To ensure the assumption of independence of observations, all multiple births were excluded and assessment of clustering at household level was carried out and the assumption of independence was upheld. Collinearity between all variables and models fit with and without adjustment were checked using Pearson’s correlation matrix.

All analyses were conducted in Stata version 11 and results were presented in the form of tables and summary statistics in odds ratios (OR), with 95 % confidence intervals (CI) and p-values.

## Results

### Background characteristics

Tables 1 and 2 provide the descriptive information on the demographic and socioeconomic characteristics of 6777 mothers whose babies were included in the study. The average age of the mothers is 28 years with a standard deviation of ±7. The majority of the mothers (73.03 %) were of the Ga-Dangme ethnic group and 91.34 % were Christians. While petty trading was stipulated as the profession for 30.18 % of the women whose babies were studied, 22.86 % were unemployed. Farmers, students, and artisans formed 17.12, 13.89 and 12.42 % of the respondents respectively. Other categories of occupation contributed smaller proportions. While a significant proportion of the mothers (34.59 %) were educated up to junior high level, about 30 % of them had primary education and 26.83 % had no education. Only 8.20 % of the mothers detailed senior high school level of education and above as their level of education. About half of the mothers (49.75 %) described their marital status as cohabiting, 28.28 % were single, 20.04 % were married and 1.95 % were divorced/separated. Of the women whose babies were studied, 29.82 % had parity of more than three, 26.59 % had parity of one, 24.64 % had parity of two and 18.95 % had parity of three. Most of the women (49.23 %) used charcoal as cooking fuel and 41.64 % used wood. Liquefied petroleum gas (LPG) contributed to only 8.84 % of the cooking fuel. Majority of the mothers (91.12 %) received IPTp during their pregnancy and 81.38 % had at least four ANC visits during pregnancy. More than half of the babies born during the study period were male (52.24 %). While 40.21 % of the babies born during the study period were not weighed, 55.05 % weighed ≥ 2.5 kg and 4.74 % weighed < 2.5 kg. The results revealed that the incidence of LBW between 2011 and 2013 is 8.72, 7.04 and 7.52 % respectively.

**Table 1:** Socio-demographic characteristics of the study participants

Characteristics	Frequency ^a^	Proportion (%)
Age group		
< 20	769	11.35
20–24	1592	23.49
25–29	1841	27.17
30–34	1411	20.82
34+	1164	17.18
Mean = 27.83 (SD = 6.97)		
Ethnicity		
Ga-Dangme	4949	73.03
Akan	357	5.27
Ewe	1054	15.55
Northern	363	5.36
Others	54	0.8
Religion		
Christianity	6190	91.34
Islamic	411	6.06
Traditional	102	1.51
Others	74	1.05
Occupation		
Unemployed	1549	22.86
Farmer	1160	17.12
Artisan	842	12.42
Trader	2045	30.18
Civil Servant	109	1.61
Student	941	13.89
Others	131	1.93
Level of education		
No Education	1818	26.83
Primary	2055	30.32
Junior School Level	2344	34.59
SHS and above	556	8.20
Others	4	0.06
Marital status		
Single	1888	28.28
Married	1338	20.04
Separated/Divorced	129	1.93
Cohabiting	3321	49.75
Parity		
Parity1	1802	26.59
Parity2	1670	24.64
Parity3	1284	18.95
Parity3+	2021	29.82
Socio economic status		
Poorest	1362	20.10
Poorer	1349	19.91
Poor	1356	20.01
Less Poor	1355	19.99
Least Poor	1355	19.99

*n* = 6777; *SD* = Standard Deviation ^a^ number of respondents across some categories may not add up to 6777 due to missing data

**Table 2:** Socio-demographic characteristics of the study participants

Characteristics	Frequency ^a^	Proportion (%)
Cooking fuel		
Gas	532	8.84
Charcoal	2964	49.23
Wood	2507	41.64
Others	18	0.30
Have you received IPTp		
Yes	6157	91.12
No	600	8.88
Number of ANC visits		
Less than four visit	1249	18.62
At least four visit	5460	81.38
Infant’s gender		
Female	3237	47.76
Male	3540	52.24
Birth-weight		
Birth weight < 2.5 kg	321	4.74
Birth weight ≥ 2.5 kg	3731	55.05
Not weighed	2725	40.21

*n* = 6777; *SD* = Standard Deviation ^a^ number of respondents across some categories may not add up to 6777 due to missing data

### Univariable and multivariable analysis

The association between the dependent and independent variables is shown in Table 3. Increasing maternal age was significantly associated with the likelihood of a mother giving birth to a baby that weighed ≥2.5 kg. Women aged 20–24, 25–29, 30–34 years were more than twice more likely to have babies who weighed ≥2.5 kg compared to those aged less than 20 years (OR:2.32, 95 % CI:1.65–3.26, OR:2.73, 95 % CI:1.96–3.79, OR:2.87, 95 % CI:2.06–4.01). Mothers aged 30 years and above were more than three times more likely to have babies who weighed ≥2.5 kg. After adjusting for occupation, parity, socioeconomic status and gender of infant, maternal age is still significantly associated with birth weight such that mothers who were 20 years and older were more than two times likely to have babies who weighed ≥2.5 kg compared to those aged <20 years.

**Table 3:** Crude and adjusted odd ratios of determinates of birth weight

	Crude		Adjusted^b^	
Characteristics	OR	*P*-values (95 % CI)	OR	*P*-value (95 % CI)
Age group				
< 20	1.00		1.00	
20–24	2.32	<0.001(1.65–3.26) ^a^	2.18	<0.001(1.53–3.10) ^a^
25–29	2.73	<0.001(1.96–3.79) ^a^	2.34	<0.001(1.62–3.39) ^a^
30–34	2.87	<0.001(2.06–4.01) ^a^	2.20	<0.001(1.48–3.28) ^a^
34+	3.59	<0.001(2.56–5.04) ^a^	2.52	<0.001(1.67–3.82) ^a^
Marital status				
Single	1.00			
Married	1.16	0.157(0.94–1.14)		
Separated/Divorced	0.89	0.690(0.51–1.55)		
Cohabiting	1.14	0.141(0.92–1.55)		
Level of education				
No Education	1.00			
Primary	0.92	0.451(0.75–1.14)		
Junior High School	1.12	0.239(0.93–1.55)		
Senior High School & above	1.19	0.184(0.92–1.55)		
Occupation				
Unemployed	1.00		1.00	
Farmer	1.33	0.022(1.04–1.70) ^a^	1.10	0.466(0.85–1.42)
Artisan	1.31	0.031(1.03–1.67) ^a^	1.23	0.115(0.95–1.58)
Trader	1.23	0.050(1.00–1.50)	1.04	0.687(0.84–1.29)
Civil Servant	1.82	0.012(1.14–2.90) ^a^	1.77	0.021(1.09–2.87) ^a^
Student	0.92	0.554(0.71–1.20)	1.31	0.068(0.98–1.74)
Others	1.40	0.197(0.84–2.35)	1.43	0.184(0.84–2.41)
Parity				
Parity1	1.00		1.00	
Parity2	1.48	<0.001(1.20–1.83) ^a^	1.30	0.026(1.03–1.63) ^a^
Parity3	1.46	<0.001(1.16–1.82) ^a^	1.23	0.116(0.95–1.59)
Parity3+	2.12	<0.001(1.74–2.59) ^a^	1.81	<0.001(1.38–2.35) ^a^
Socio economic status				
Poorest	1.00		1.00	
Poorer	1.30	0.033(1.02–1.65) ^a^	1.30	0.040(1.01–1.66) ^a^
Poor	1.03	0.795(0.81–1.31)	1.03	0.796(0.81–1.32)
Less Poor	0.93	0.516(0.73–1.17)	0.93	0.578(0.74–1.19)
Least Poor	1.27	0.033(1.02–1.58) ^a^	1.24	0.064(0.99–1.55)
Infant’s gender				
Female	1.00		1.00	
Male	1.52	<0.001(1.32–1.76) ^a^	1.56	<0.001(1.35–1.81) ^a^
Have you receive IPTp				
Yes	1.00			
No	1.15	0.333(0.87–1.52)		
Number of ANC visits				
Less than four visit	1.00			
At least four visit	1.15	0.206(0.92–1.44)		
Cooking fuel				
Gas	1.00			
Charcoal	0.82	0.099(0.65–1.04)		
Wood	0.95	0.682(0.74–1.22)		
Others	0.28	0.222(0.03–2.18)		

*OR* Odd Ratio, ^a^statistically significant. *CI* Confidence Interval. ^b^Correct classification rate of the model = 75.96 %

Marital status, level of education, IPTp intake and number of ANC visits were not significantly associated with birth weight. Mothers who were farmers, artisans or civil servants were 33, 31, 82 % respectively more likely to have babies who weighed ≥2.5 kg (OR:1.33, 95 % CI:1.04–1.70, OR:1.31, 95 % CI:1.03–1.67, OR:1.82, 95 % CI:1.14–2.90) compared to unemployed mothers. After adjusting for parity, socioeconomic status and infant gender, civil servants were 77 % more likely to have babies who weighed ≥2.5 kg (OR:1.77, 95 % CI:1.99–2.87) compared with those mothers who were unemployed.

While women with parity 2 and 3 were 48 % and 46 % more likely to have babies who weighed ≥2.5 kg respectively (OR: 1.48, 95 % CI: 1.20–1.83, OR: 1.46, 95 % CI: 1.16–1.82), those with parity higher than three were more than two times more likely to have babies weighing ≥2.5 kg (OR: 2.12, 95 % CI: 1.74–2.59) compared to those with parity one. In the presence of other explanatory variables, women with parity two and more than three were 30 % and 81 % more likely to have babies weighing ≥2.5 kg respectively (OR:1.30, 95 % CI:1.03–1.63, OR:1.81, 95 % CI:1.38–2.35) compared to those with parity one.

Mothers within the poorer socioeconomic status category were 30 % more likely to have babies who weighed ≥2.5 kg (OR: 1.30, 95 % CI: 1.02–1.65) compared to those in the poorest category. Those women from least poor households were 27 % more likely to have babies weighed ≥2.5 kg (OR: 1.27, 95 % CI: 1.02–1.58) compared to those from the poorest households. After adjusting for other explanation variables, mothers from poorer households were 30 % more likely to have babies who weighed ≥2.5 kg (OR: 1.30, 95 % CI: 1.01–1.66).

Male infants were 52 % more likely to weigh ≥2.5 kg at birth (OR: 1.52, 95 % CI: 1.32–1.76) compared to females and this was statistically significant after adjusting for other explanatory variables (OR: 1.56, 95 % CI: 1.35–1.81).

## Discussion

This study sought to examine the determinants of birth weight and the incidence of LBW in southern rural Ghana using secondary data from DHDSS from 1st January 2011 to 31st December 2013. The incidence of LBW in this population-based study is a little lower than that of Ghana MICS which found a LBW prevalence of 9.1 % and 11 % in 2006 and 2011 respectively [4, 36]. Our study revealed that having infant birth weight ≥ 2.5 kg is highly associated with socioeconomic status of women household, gender of infant, parity, occupation and maternal age.

The findings show a strong association between birth weight and socioeconomic status which is consistent with other studies which showed that higher socioeconomic status reduced the risk of LBW [40–42]. This shows that poverty is an important determinant of birth weight as shown in other contexts [43–45]. Low birth weight could be due to poor maternal nutritional intake among mothers with lower socioeconomic status as found in other studies [46, 47].

The findings suggested that a mother’s level of education is not associated with birth weight which is consistent with some studies which reported that birth weight is not statistically significant with maternal level of education [48, 49], it however contrasts the findings of other studies [50, 51] which found an association between birth weight and the mother’s level of education.

The results of this study also indicate that the gender of infants is highly associated with birth weight, male infants are more likely to experience birth weight ≥2.5 kg than female infants. Similar findings have been reported in other studies [52–54].

The results also indicated that mothers aged below 20 years had significantly greater chances of delivering LBW babies than the age group above 20 years which corresponds with the findings in other studies [55, 56]. The strong association between maternal age and birth weight found by our study is consistent with the findings of Vahdaninia et al.(2008) [57]. This could be due to the fact that, adolescent mothers are still in the process of biological growth and may not be physically and emotionally mature enough to know the importance of child bearing, self-care and good nutrition during pregnancy [46]. Other studies [58, 59] found a significant correlation between mother’s antenatal care visits and birth weight which contradicts the findings of our study.

The association between birth weight and parity has been established by other studies [41, 60] and our findings are consistent with the findings of these. The results of our study showed a significant relationship between maternal occupation and infant birth weight. These findings are contrary to findings of other studies which found no association between maternal occupation and infant birth weight [61–64].

### Limitations

Although this study offers some advantages such as the large sample size and quality of data, it also has important limitations. Other important variables that have been shown to affect birth weight, such as mother’s weight gain during pregnancy, maternal history of disease, gestational age [65, 66], were not available in the secondary data used for this study. These factors and the high proportion of babies not weighed at birth due to deliveries outside health facilities might have provided additional information about the determinants of birth weight. The generalization of these findings beyond the study districts may be unlikely because the DHDSS covers only two districts.

## Conclusion

This population based study reveals important information about the determinants of birth weight of babies born in southern rural Ghana. Maternal age, occupation, parity, socioeconomic status and infant’s gender, appear to be significant determinants of birth weight.

Low birth weight is a significant public health problem and as multiple factors are associated with it, it requires a more holistic and multisectorial approach for its reduction. Interventions targeting high-risk women needs to be implemented to provide better health care services to all antenatal teenagers and women with high parity. Early registrations of pregnancy should be promoted so as to detect the presence of any high‑risk factors as early as possible during the pregnancy. Health promotion in this regard should be targeted towards the high‑risk women. Targeting social interventions to improve the socioeconomic status of women from poor households may help reduce the incidents of LBW. Further research in this area is needed to explore and explain the influence of other important variables on birth weight.

## Abbreviations

ANC, antenatal care; CDC, Centres for Disease and Control; CI, confidence intervals; DHDSS, Dodowa Health and Demographic Surveillance System; DHRC, Dodowa Health Research Centre; ELBW, extremely low birth weight; GDHS, Ghana Demographic Health Survey; HBW, high birth weight; LBW, low birth weight; LPG, liquefied petroleum gas; MDG, Millennium Development Goal; MICS, Multiple Indicator Cluster Survey; NBW, normal birth weight; OR, odd ratio; PCA, principal component analysis; VLBW, very low birth weight
